# Raindrop energy-powered autonomous wireless hyetometer based on liquid–solid contact electrification

**DOI:** 10.1038/s41378-022-00362-6

**Published:** 2022-03-14

**Authors:** Chaoqun Xu, Xianpeng Fu, Chengyu Li, Guoxu Liu, Yuyu Gao, Youchao Qi, Tianzhao Bu, Yuanfen Chen, Zhong Lin Wang, Chi Zhang

**Affiliations:** 1 Center on Nanoenergy Research, School of Physical Science & Technology, School of Mechanical Engineering, Guangxi University, Nanning, 530004 China; 2 CAS Center for Excellence in Nanoscience, Beijing Key Laboratory of Micro-nano Energy and Sensor, Beijing Institute of Nanoenergy and Nanosystems, Chinese Academy of Sciences, Beijing, 101400 China; 3 School of Nanoscience and Technology, University of Chinese Academy of Sciences, Beijing, 100049 China; 4 School of Material Science and Engineering, Georgia Institute of Technology, Atlanta, GA 30332 USA

**Keywords:** Electrical and electronic engineering, Environmental, health and safety issues

## Abstract

Triboelectric nanogenerators (TENGs) can directly harvest energy via solid–liquid interface contact electrification, making them very suitable for harvesting raindrop energy and as active rainfall sensors. This technology is promising for realizing a fully self-powered system for autonomous rainfall monitoring combined with energy harvesting/sensing. Here, we report a raindrop energy-powered autonomous rainfall monitoring and wireless transmission system (R-RMS), in which a raindrop-TENG (R-TENG) array simultaneously serves as a raindrop energy harvester and rainfall sensor. At a rainfall intensity of 71 mm/min, the R-TENG array can generate an average short-circuit current, open-circuit voltage, and maximum output power of 15 μA, 1800 V, and 325 μW, respectively. The collected energy can be adjusted to act as a stable 2.5 V direct-current source for the whole system by a power management circuit. Meanwhile, the R-TENG array acts as a rainfall sensor, in which the output signal can be monitored and the measured data are wirelessly transmitted. Under a rainfall intensity of 71 mm/min, the R-RMS can be continuously powered and autonomously transmit rainfall data once every 4 min. This work has paved the way for raindrop energy-powered wireless hyetometers, which have exhibited broad prospects in unattended weather monitoring, field surveys, and the Internet of Things.

## Introduction

Rain is a major source of fresh water for human beings and other terrestrial organisms^1–8^. The rain gauge is a significant device for rainfall monitoring that is usually employed in unattended environments and requires a long service life. To date, the working principle of conventional rain gauges mainly includes siphoning, weighing, and tipping bucket types, which often have complex mechanical structures^9,10^. Moreover, the electronic devices in rain gauges are mainly powered by short-lived batteries, which greatly increases maintenance costs and environmental pollution. Therefore, harvesting raindrop energy^11,12^ from the ambient environment to power rain gauges is an alternative method for realizing sustainable rainfall monitoring^13,14^.

Since the triboelectric nanogenerator (TENG) based on Maxwell’s displacement current was proposed in 2012^15–17^, it has been developed as a promising technology for rain energy harvesting^18–20^ and active sensing^21–23^. For rain energy harvesting, TENGs can directly convert raindrop energy into electricity based on solid–liquid interface contact electrification^24–30^, which can provide sustainable micro/nanopower sources for distributed electronic devices^26,27^. For rainfall monitoring, a TENG can directly convert rainfall information into an electrical signal without other complex mechanical structures. However, the signal processing and transmission of this kind of active sensor still need an external power supply^28–37^. If a raindrop-TENG (R-TENG) can be simultaneously used as a rainfall sensor and energy harvester and the signal processing and transmission of the rainfall sensor can be powered by the energy harvester, this would very promising for realizing a fully self-powered sensing system for autonomous rainfall monitoring^38^.

Herein, we report a raindrop energy-powered autonomous rainfall monitoring and wireless transmission system (R-RMS), in which an R-TENG array simultaneously serves as the raindrop energy harvester and rainfall sensor. At a rainfall intensity of 71 mm/min, the R-TENG array can generate an average short-circuit current, open-circuit voltage, and maximum output power of 15 μA, 1800 V, and 325 μW, respectively. Through the regulation of the EMM, the collected energy can be turned into a stable 2.5 V direct-current (DC) output for the whole system. Additionally, the R-TENG array acts as a rainfall sensor, in which the output signal can be calculated and measured data can be wirelessly transmitted. Under a rainfall intensity of 71 mm/min, the R-RMS can be continuously powered and autonomously transmit rainfall data every 4 min. This work has paved the way for raindrop energy-powered wireless hyetometers that have exhibited broad prospects in unattended weather monitoring, field surveys, and the Internet of Things^36,39,40^.

## Results and discussion

### Characterization of the R-RMS

**Fig. 1:**
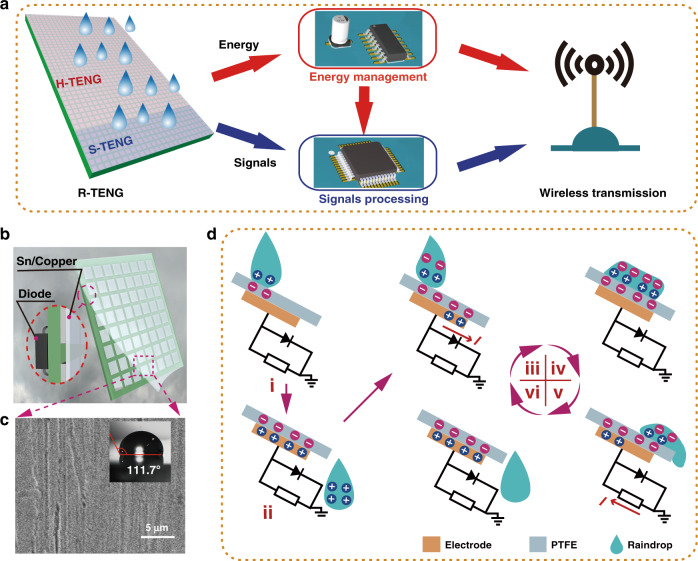
Structure and working mechanism of the raindrop-powered autonomous rainfall monitoring and wireless transmission system (R-RMS). **a** Framework for the R-RMS. **b** Structural design of the fabricated R-TENG. **c** SEM image of the treated PTFE surface and raindrop contact angle. **d** Working principle of the R-TENG unit

Figure 1 shows the structure and working mechanism of the raindrop energy-powered autonomous R-RMS. As shown in Fig. 1a, the R-RMS consists of an R-TENG, an energy management module (EMM), a signal processing module (SPM), and a wireless transmitter. The R-TENG is composed of a harvesting energy TENG (H-TENG) and a signal TENG (S-TENG) for raindrop energy harvesting and rainfall monitoring, respectively. For raindrop energy harvesting, the H-TENG can convert the raindrop energy into electrical energy. Through the EMM, a steady 2.5 V DC voltage can be provided to the SPM and wireless transmitter. For rainfall monitoring, the S-TENG converts rainfall information into an electrical signal. Then, the electrical signal is regulated and analyzed by the SPM. Finally, the measured rainfall data are wirelessly sent to the remote receiving terminal through the wireless transmitter. With the continuous raindrop energy, the R-RMS can sustainably and autonomously transmit measurement data of rainfall.

As shown in Fig. 1b, the R-TENG array is fabricated in single-electrode mode with a diode in each unit for rectification. The copper electrode array is fabricated by printed circuit board (PCB) technology. A polytetrafluoroethylene (PTFE) film is attached to the surfaces of the copper electrode. To improve the hydrophobicity of the PTFE surface, the surface of the PTFE film is modified at the microstructural level. The details of the fabrication process are given in the “Materials and methods”. Figure 1c shows an SEM image of the surface of the PTFE film and a photo of the contact angle of the liquid drop. The contact angle between the raindrop and the PTFE surface reaches 111.7° after surface modification, so the raindrop can roll off the PTFE surface smoothly.

The working mechanism of the R-TENG is based on contact electrification and electrostatic induction, as shown in Fig. 1d. The basic unit of the R-TENG consists of a PTFE film, a copper electrode, and a diode for the external load resistance. At the initial state, the raindrop falls and contacts the PTFE film, as shown in Fig. 1d. (i) Electrons are transferred from the raindrop to the PTFE surfaces due to the different electronegativities, and then equal amounts of positive and negative charge can be induced on the raindrop and PTFE surfaces, respectively. As the raindrop leaves the PTFE film, a positive charge is generated at the copper electrode to achieve a new electrostatic balance. After a certain working cycle, the negative charge on the PTFE surface can reach saturation, as shown in Fig. 1d. (ii) Figure S1 shows the potential distribution when the raindrop has a maximum contact area with the PTFE film. When another raindrop falls and contacts the PTFE surface, an electrical double layer is generated on the contact surface of the raindrop to maintain electrical neutrality^41^, rather than electrons transferring between the raindrop and the PTFE, as shown in Fig. 1d. (iii) In this state, the copper electrode is at a relatively high potential. Equivalently, a forward bias voltage is applied to the diode, making the diode turn on with a very small resistance^42^. Because the resistance of the diode is much smaller than the load resistance at this time, the current mainly flows through the diode until a new electrostatic balance is reached (Fig. 1d (iv)).

As the raindrop leaves the PTFE film, the copper electrode is at a relatively lower potential. The diode is a snap-off diode with a larger resistance due to the reverse bias voltage. The current can pass through the load resistance, as shown in Fig. 1d (v). Once the raindrop completely leaves the PTFE film, as shown in Fig. 1d (vi), electrostatic balance is achieved again. From the above analysis, the electricity-generating process is a unidirectional current through the load resistance. Figure S2 shows the current and charge characteristics of a raindrop on the R-TENG.

### Electrical output and energy management characteristics of the H-TENG array

**Fig. 2:**
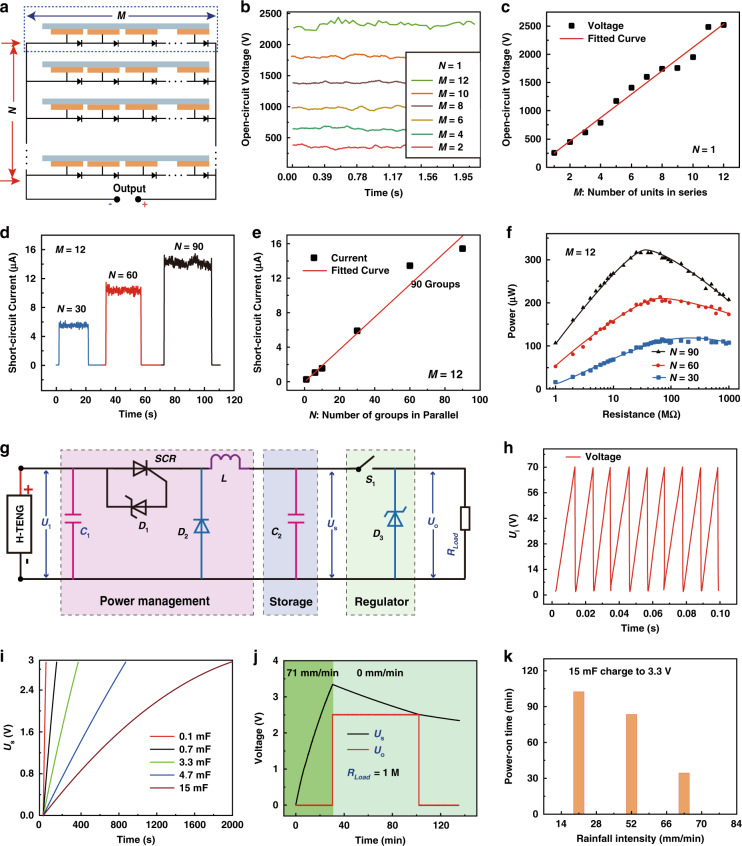
Electrical output and energy management characteristics of the H-TENG array. **a** Schematic diagram of the H-TENG array (*M* × *N*). The following data are measured at a rainfall intensity of 71 mm/min if not specified. **b** Open-circuit voltage waveforms of the H-TENG units in series (*N* = 1). **c** Relationship between the open-circuit voltage and *M* (*N* = 1). **d** Short-circuit current waveforms of the H-TENG groups in parallel (*M* = 12). **e** Relationship between the short-circuit current and *N* (*M* = 12). **f** Output power of the H-TENG with different resistances (*M* = 12; *N* = 30, 60, 90). **g** Schematic circuit diagram of the EMM for the H-TENG. **h** Voltage waveform of the H-TENG with the EMM (*C*_2_ = 15 mF). **i** Charging characteristics of different capacitors (*C*_2_). **j** The storage and regulation voltage waveforms of the EMM with 1 MΩ load resistance. **k** Charging time for a 15 mF capacitor to 3.3 V with different rainfall intensity rates

The electrical output and energy management characteristics of the H-TENG array are shown in Fig. 2. Figure 2a depicts a detailed schematic diagram of the electrical connections of the H-TENG, in which *M* units are connected in series and *N* groups are connected in parallel. The H-TENG array is characterized at a rainfall intensity of 71 mm/min if not specified. Figure 2b and Figure S3 show the output open-circuit voltage waveforms of the H-TENG with different values of *M* ranging from 1 to 12 (*N* = 1). As shown in Fig. 2c, the peak-to-peak value of the open-circuit voltage (Vpp) increases with increasing *M*. Vpp increases from 255 to 2528 V when *M* increases from 1 to 12. For *N* = 1, the output waveforms of the short-circuit current with different values of *M* are shown in Figure S4. Figure 2d indicates the output waveforms of the short-circuit current of the H-TENG with different *N* values ranging from 1 to 90 (*M* = 12). The peak value of the short-circuit current (*I*_SC_) increases with increasing *N*, as shown in Fig. 2e. *I*_SC_ increases from 0.255 to 15.427 μA when *N* increases from 1 to 90. The generated electricity can continuously illuminate 40 LEDs, as shown in Video S1. The impedance characteristics of the H-TENG at different values of *N* (*M* = 12) are systematically studied, as shown in Fig. 2f. At each *N*, the instantaneous power initially rises and then drops, achieving a maximum value at the matching resistance. The maximum value of the instantaneous power increases with increasing *N*, while the matching resistance decreases. When *N* increases from 30 to 90, the maximum values of the instantaneous power increase from 114.6 to 316.57 μW, and the matching resistance decreases from 70 to 25 MΩ. The output current and voltage for a series of resistances are shown in Fig. S5.

The output electrical energy of the H-TENG is regulated with the EMM to provide a steady 2.5 V DC voltage for the SPM and transmitter. Figure 2g shows the schematic circuit diagram of the EMM, which consists of a power management circuit (PMM), a storage capacitor, and a regulator. The PMM is composed of a capacitor *C*_1_ = 2.2 μF, a serial silicon controlled rectifier (SCR)^43–47^, a Zener diode *D*_1_, an inductor *L* = 2.4 mH, and a parallel freewheeling diode *D*_2_ for adjusting the voltage and impedance of the H-TENG. When the voltage *U*_i_ of capacitor *C*_1_ achieves the stabilized voltage value of *D*_1_, the SCR is turned on, and the stored energy in capacitor *C*_1_ is temporarily stored in inductor *L* with the snap-off diode *D*_2_. When the SCR is turned off, the temporarily stored energy in inductor *L* is transferred to the storage capacitor *C*_2_ through the opened *D*_2_. With the continuous operation of the SCR, the output energy of the H-TENG can be efficiently stored in *C*_2_. The charging efficiency can be more dramatically improved with the EMM than with the rectifier. As shown in Fig. S6, the stored voltage *U*_s_ can reach 10 V with the EMM after 9.7 s of charging, while direct charging takes 33 s. The stored voltage *U*_s_ can be controlled by the regulator, which consists of switch *S*_1_ and voltage stabilizer *D*_3_. Once *U*_s_ achieves a certain value, *S*_2_ is switched on, and *U*_o_ is pulled up from zero to a steady voltage. Based on the EMM, the output voltage of the H-TENG can be transferred into a steady DC voltage and power for the SPM and transmitter.

Figure 2h shows the voltage waveform of *U*_i_ with the continuous operation of the SCR. When the potential difference between *U*_i_ and *U*_o_ reaches 70 V, the stored energy in capacitor *C*_1_ is transferred into inductor *L* through the opened *SCR*, and the potential difference between *U*_i_ and *U*_o_ decreases from 70 to 0 V. Figure 2i shows the charging characteristics of the different capacitors with the EMM. When the capacitor is charged to 3 V, the charging times are 66, 137, 386, 868, and 1971 s for 0.1, 0.7, 3.3, 4.7, and 15 mF capacitors, respectively. Figure 2j shows the waveforms of *U*_s_ and *U*_o_ with a 1 MΩ load resistance, indicating the duration of power-on. When the H-TENG operates at 71 mm/min, *U*_s_ rises from 0 to 3.3 V over 33 min until the rain stops. During this time, *U*_o_ immediately decreases from 0 to 2.5 V when *U*_s_ exceeds 3.3 V. Even when the rain stops and *U*_s_ slowly drops due to the energy consumption of the load resistances, *U*_o_ can be stably maintained at 2.5 V for a duration of time until *U*_s_ is below 2.5 V. The power-on time with the different rainfalls is shown in Fig. 2K, which indicates that the power-on time decreases with greater rainfall intensity.

### Rainfall monitoring mechanism and characteristics of the S-TENG

**Fig. 3:**
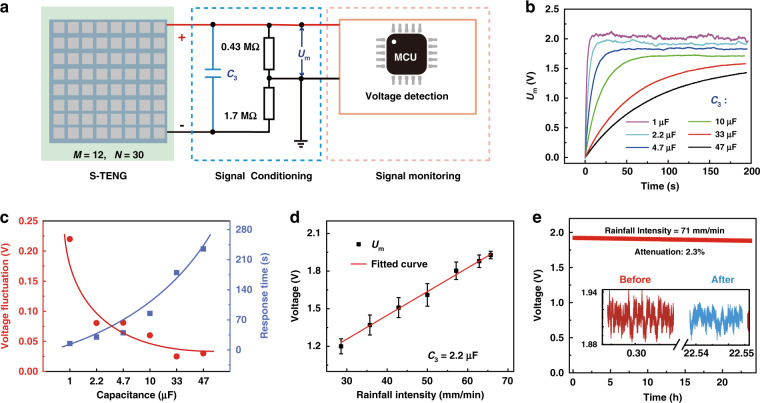
Rainfall monitoring mechanism and characteristics of the S-TENG. **a** Schematic diagram of rainfall monitoring with the S-TENG. **b** Measured voltage waveforms with different capacitors. **c** Voltage fluctuations and response times for rainfall monitoring with different capacitors. **d** Relationship between the measured voltage and flow rate of water. **e** Stability of the rainfall monitoring; inset: the measured voltage waveforms for rainfall monitoring before and after 25 h at 71 mm/min

Figure 3 indicates the rainfall monitoring mechanism and characteristics of the S-TENG. The S-TENG is connected to the SPM to monitor rainfall. As raindrops fall onto the S-TENG, the rainfall can be measured by the SPM according to the voltage output from the S-TENG. Figure 3a shows a schematic diagram of rainfall monitoring by the S-TENG and SPM. The SPM is composed of a signal conditioning circuit and microprogrammed control unit (MCU). The signal conditioning circuit consists of a capacitor *C*_3_ and two parallel resistors *R*_1_ = 0.43 MΩ and *R*_2_ = 1.7 MΩ, which are employed to convert the output voltage signals of the S-TENG into a detectable voltage signal for the MCU. The MCU is used to analyze the rainfall information from the signal conditioning circuit. When raindrops fall onto the S-TENG, the voltage of capacitor *C*_3_ can achieve a stable value so that the input energy from the S-TENG and energy consumed by resistors *R*_1_ and *R*_2_ are balanced. At the same time, the voltage *U*_m_ across resistor *R*_1_ can reach a stable value. With different rainfall intensities, *U*_m_ can reach different stable values. Therefore, the rainfall can be obtained by the MCU according to the voltage *U*_m_. The voltage waveforms of *U*_m_ with different capacitances of *C*_3_ are summarized in Fig. 3b. With a smaller capacitance, *U*_m_ can reach a stable value faster but with a larger fluctuation. Figure 3c shows the voltage fluctuation and response time for rainfall monitoring with different capacitances of *C*_3_. The voltage fluctuation decreases with increasing capacitance, while the response time increases. Considering both the voltage fluctuation and response time, *C*_3_ = 2.2 μF is chosen in the SPM. Figure 3d indicates the voltage measured for different rainfall intensities. The measured voltage has a strong linear relationship with the rainfall intensity, demonstrating that it performs well for rainfall sensing. The sensitivity of the R-RMS can reach 0.239 V/(mm/min). The R-RMS has excellent measurement accuracy; the maximal standard deviation is 3.17 mm/min with 10 repeated measurements at each rainfall level. The stability of the R-RMS is also investigated, as shown in Fig. 3e. *U*_m_ decreases 2.3% after 25 h of continuous operation at 71 mm/min.

### Application of autonomous and sustainable rainfall monitoring and wireless data transmission

**Fig. 4:**
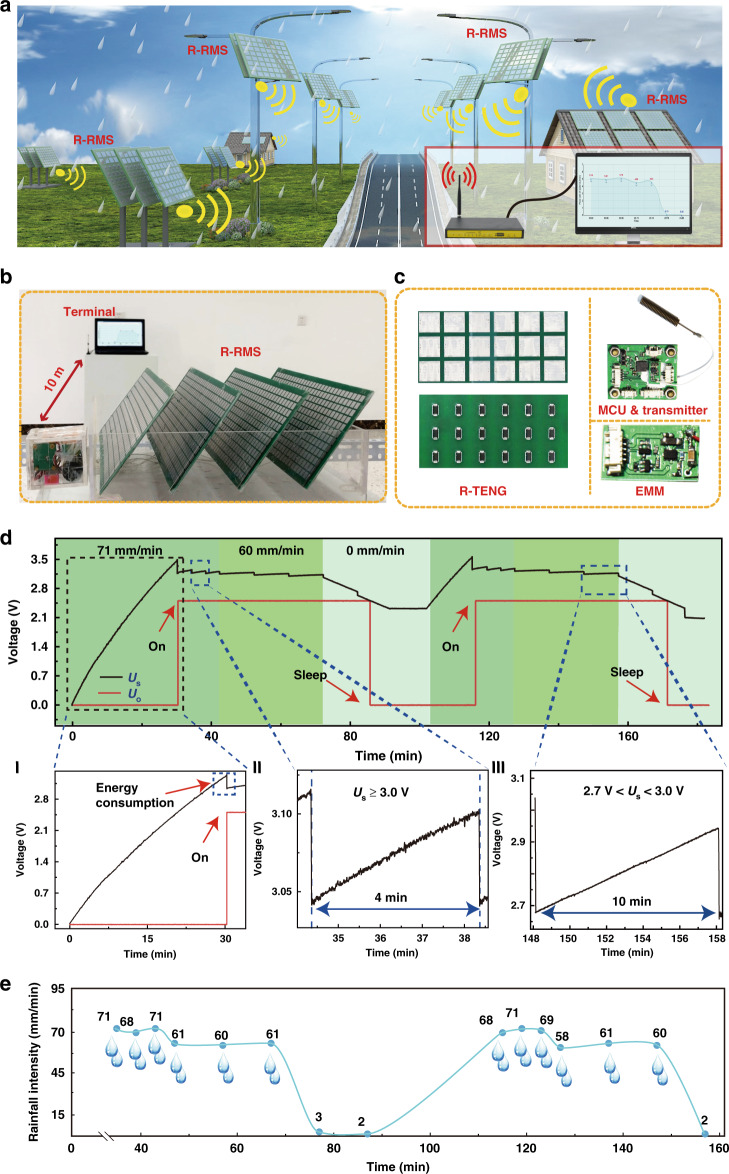
Application for autonomous and sustainable rainfall monitoring and wireless data transmission. **a** Outdoor application prospect sketch of the R-RMS. **b** Demonstration of the R-RMS in a simulated rainy environment. **c** Optical images of the R-TENG, EMM, and MCU and wireless module. **d** The voltage variation of the capacitor connected with the EMM for several consequent transmitting cycles. **e** Rainfall data acquired by the wireless receiving terminal

By coupling raindrop energy harvesting and rainfall sensing, the R-RMS can realize autonomous and sustainable wireless rainfall sensing and data transmission, as shown in Fig. 4. The EMM, SPM, and transmitter are integrated with the R-TENG and indicate a potential application of the R-RMS for unattended rainfall monitoring with long service life, as shown in Fig. 4a. Figure 4b indicates a simulated rainy environmental experiment for the R-RMS. The R-RMS is driven by artificial and adjustable rainfall, and a display terminal is employed as the receiver 10 m away. Optical photos of the R-TENG, EMM, MCU, and wireless transmitter are shown in Fig. 4c. The voltage waveforms of *U*_s_ and *U*_o_ when the R-RMS is stimulated by rain at different rainfall intensity rates are shown in Fig. 4d. In the initial state, both *U*_s_ and *U*_o_ are 0 V. When the R-RMS is initiated by rain at 71 mm/min, *U*_s_ increases to 3.3 V in 33 min, as shown in Fig. 4d(I). Meanwhile, *U*_o_ is stabilized at 2.5 V, and *U*_S_ immediately drops to 3.02 V, indicating that 13.27 mJ of energy is consumed by the activation of the SPM. Video S2 shows the activation process of the R-RMS. After activation, *U*_o_ can be maintained at 2.5 V regardless of the voltage variation of *U*_S_. The sampling and wireless transmitting period of the rainfall data are controlled by the MCU according to the *U*_S_, which is also periodically monitored by the MCU to determine the rest energy. The data are transmitted with an energy consumption of 3.3 mJ. When the R-RMS is driven by rainfall at 71 mm/min, *U*_S_ can remain above 3.0 V to balance raindrop energy harvesting and power consumption in the R-RMS. The rainfall data are sampled and transmitted every 4 min, as shown in Fig. 4d(II). However, when the R-RMS is excited by rainfall at 58 mm/min, *U*_S_ can remain above 2.7 V, and the cycle extends to 10 min, as shown in Fig. 4d(III). Once the rainfall decreases to 0 mm/min, the energy stored in capacitor *C*_2_ is gradually consumed. When *U*_S_ decreases to 2.5 V, *U*_o_ returns to 0 V, and the R-RMS switches into the sleep state. If the R-RMS is driven by rainfall and *U*_S_ reaches 3.3 V again, the R-RMS awakens, and *U*_o_ recovers to 2.5 V. As a demonstration, the rainfall data received during 150 min are summarized in Fig. 4e. The energy harvested by the H-TENG can meet the energy consumption requirements for processing and transmitting the sensing signal from the S-TENG. With continuous rainfall, the R-RMS can sustainably and autonomously provide monitoring data by wireless communication, which has exhibited broad prospects in unattended weather monitoring, field surveys, and the Internet of Things.

## Conclusion

In conclusion, we demonstrated an R-RMS based on an R-TENG for simultaneous raindrop energy harvesting and rainfall monitoring. Benefiting from the contact electrification between raindrops and the PTFE film, the R-RMS array can autonomously activate by raindrop energy harvesting. At different rainfall amounts, the output characteristics of the R-RMS are investigated. The electrical energy output of the R-RMS can be used as a stable 2.5 V DC power supply for the whole system, including the SPM and data transmitter. Meanwhile, the output signal of the R-RMS can indicate rainfall through voltage measurement. Through the autonomous monitoring mechanism, the monitoring and transmission cycle can be intelligently adjusted according to the stored energy and external input power. When the rainfall is 71 mm/min, the rainfall data are automatically transmitted every 4 min within the range of 10 m. The energy collected by the R-RMS can meet the energy consumption requirements of sensor signal processing and data transmission. This work achieved a fully TENG-based self-powered sensing system and can pave the way for a raindrop energy-powered wireless hyetometer. It has broad prospects in the fields of the Internet of Things, meteorological monitoring, and geological disaster early warning.

## Experimental section

### Manufacturing of the R-TENG

The R-TENG is composed of surface-treated PTFE film, industrially processed PCB board, and diode array. Shell size: 54 mm × 34 mm. The copper electrode array is a square of 10 mm × 10 mm with a thickness of 0.16 mm and a gap of 2 mm. The 30 μm PTFE film is surface-treated and is applied to the PCB board. The bottom waterproofing is sealed by pouring a two-component epoxy resin AB adhesive.

### Characterization of the EMM

The EMM consists of a PMM, a storage capacitor, and a regulator. The PMM is composed of a capacitor *C*_1_ = 2.2 μF, a serial SCR, a Zener diode *D*_1_, an inductor *L* = 2.4 mH and a parallel freewheeling diode *D*_2_ for adjusting the voltage and impedance of the H-TENG. The stabilized voltage values of *D*_1_ and *D*_3_ are 70 V and 2.5 V, respectively.

### Characterization of the SPM

The SPM is composed of a signal conditioning circuit and MCU. The signal conditioning circuit consists of a capacitor *C*_3_ and two parallel resistors *R*_1_ = 0.43 MΩ and *R*_2_ = 1.7 MΩ, which are employed to convert the output voltage signals of the S-TENG into a detectable voltage signal for the MCU.

### Characterization and measurement

A rain shower is used to provide a continuous and speed-controllable rainfall simulation environment for the R-RMS. The hardware system of the R-RMS is based on the C8051f410 microcontroller. The data processing and forwarding are coded with a Python program based on a Raspberry Pi. The data are stored using MySQL in a workstation based on Alibaba Cloud, and the data are displayed using a website developed based on PHP that can be displayed in real-time on any mobile phone, computer, and other networked terminals.

## Supplementary information

Marked up Revised Supporting Information

Revised Supporting Information

The LEDs are continuously illuminated by the R-TENG

R-RMS simultaneously collects raindrop energy and measures rainfall for self-powered wireless rainfall monitoring and data transmission
