# Supplementary material for: Raindrop energy-powered autonomous wireless hyetometer based on liquid–solid contact electrification
Source: Microsyst Nanoeng. 2022 Mar 14;8:30. doi: 10.1038/s41378-022-00362-6 (PMC8918552; doi:10.1038/s41378-022-00362-6)
Supplement: Supplementary file 1 — Marked up Revised Supporting Information [file 41378_2022_362_MOESM1_ESM.docx]

Supporting Information

Raindrop energy powered autonomous wireless hyetometer based on liquid-solid contact electrification

*Chaoqun Xu^1,2,#^, Xianpeng Fu ^2,3,#^, Chengyu Li^1^, Guoxu Liu^2,3^, Yuyu Gao^2^, Youchao Qi^2,3^, Tianzhao Bu^2,3^, Yuanfen Chen^1,*^, Zhong Lin Wang^2,3,4,*^, and* *Chi Zhang^1,2,3,*^*.

^*^Corresponding author.

*E-mail addresses:* yuanfenchen@gxu.edu.cn (Y. F. Chen), [zlwang@gatech.edu](mailto:zlwang@gatech.edu) (Z. L. Wang), [czhang@binn.cas.cn](mailto:czhang@binn.cas.cn) (C. Zhang).

^#^These authors contributed equally to this work.

^1^ Center on Nanoenergy Research, School of Physical Science & Technology, School of Mechanical Engineering, Guangxi University, Nanning 530004, China

^2^ CAS Center for Excellence in Nanoscience, Beijing Key Laboratory of Micro-nano Energy and Sensor, Beijing Institute of Nanoenergy and Nanosystems, Chinese Academy of Sciences, Beijing 101400, China

^3^ School of Nanoscience and Technology, University of Chinese Academy of Sciences, Beijing 100049, China

^4^ School of Material Science and Engineering, Georgia Institute of Technology, Atlanta, GA 30332, United States


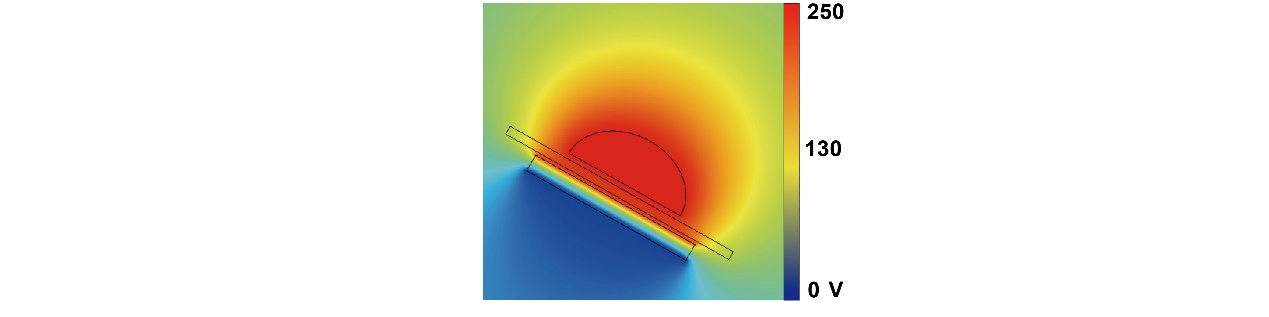


**Figure S1. Electric potential simulation of raindrop in contact with PTFE.**


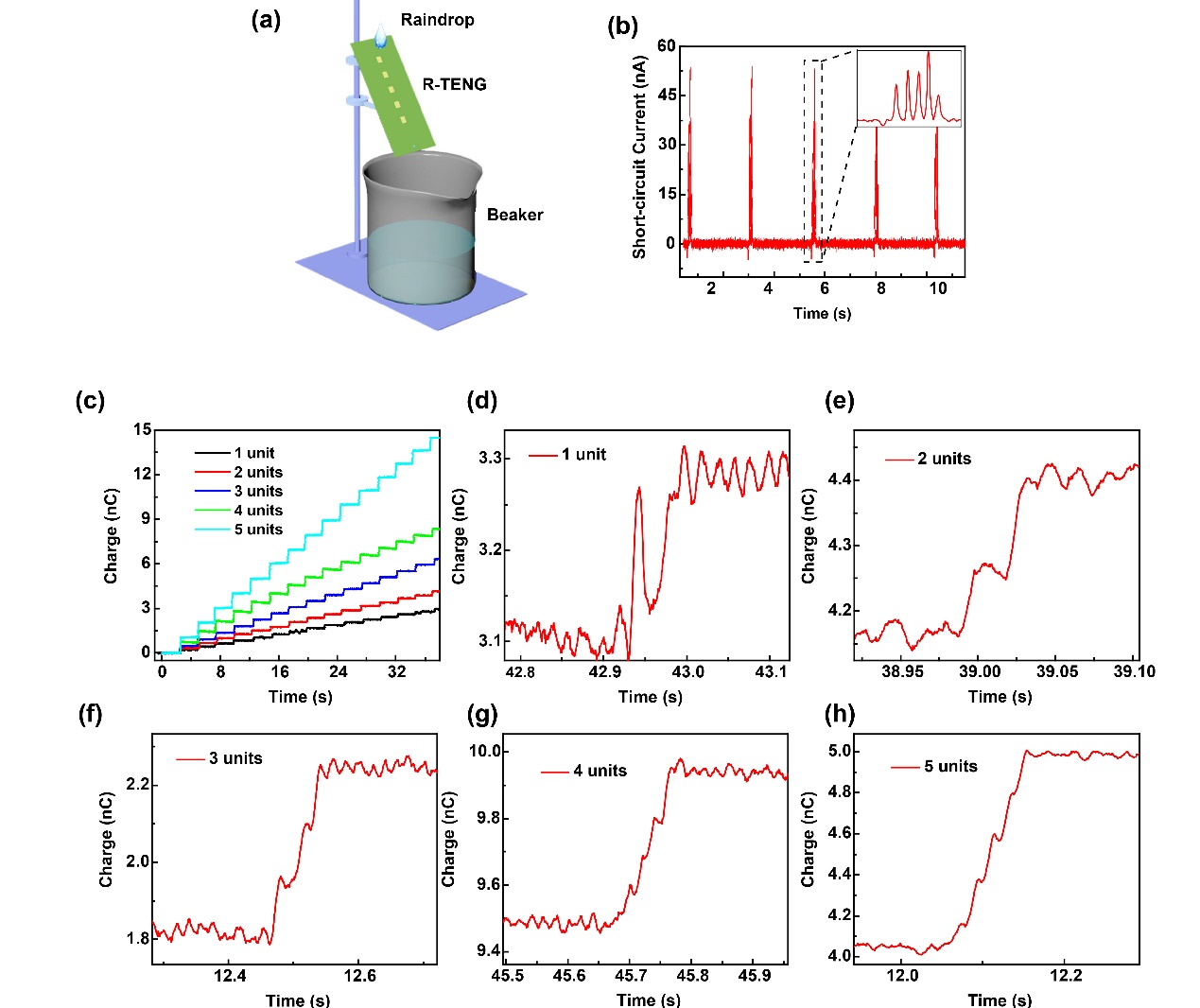
**Figure S2. Characteristics of current and charge generated by R-TENG in working state.** (a) Experimental device. (b) Short-circuit current generated by a drop of water in 5 units of R-TENG in series. (c) The charge generated by a drop of water in series with different units of R-TENG. (d, e, f, g, h) An enlarged view of the partial square of the curve in Figure (c).


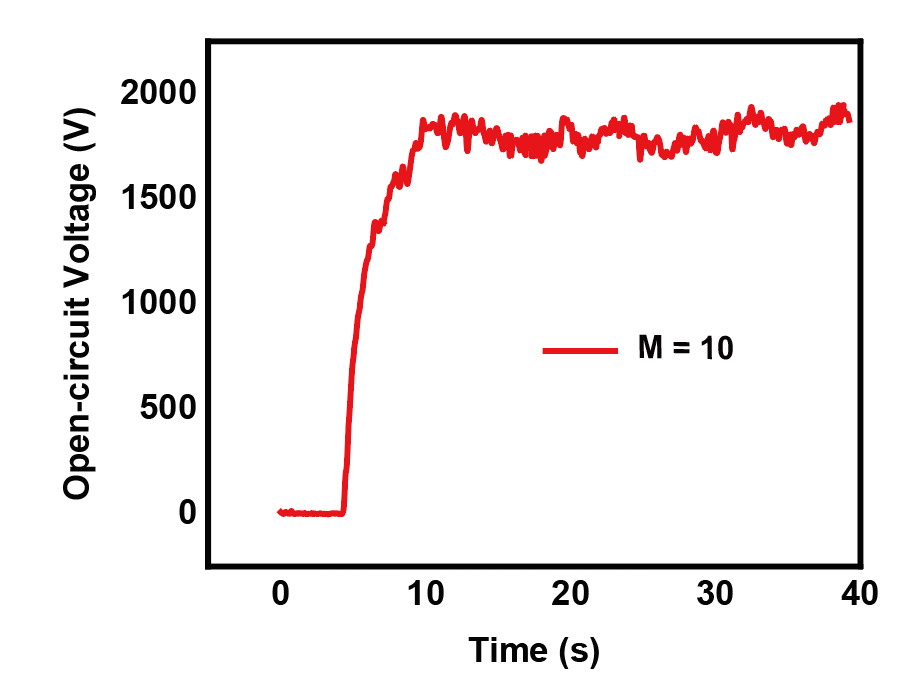


**Fig. S3. Open-circuit voltage waveforms of the H-TENG units in series (N=1, M=10).**


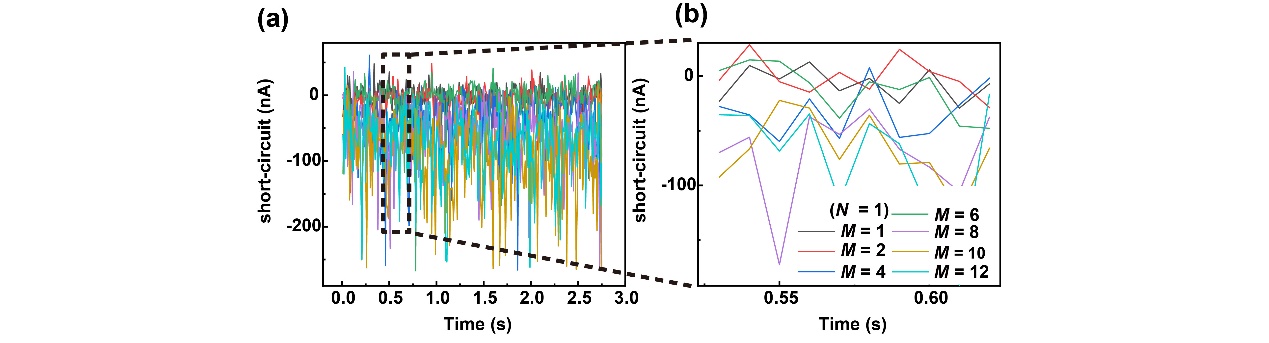


Figure S4. Short-circuit current of the R-TENG units connected in series (N=1). (a) Short-circuit current of the R-TENG units connected in series. (b) An enlarged view of the partial square of the curve in Figure (a).


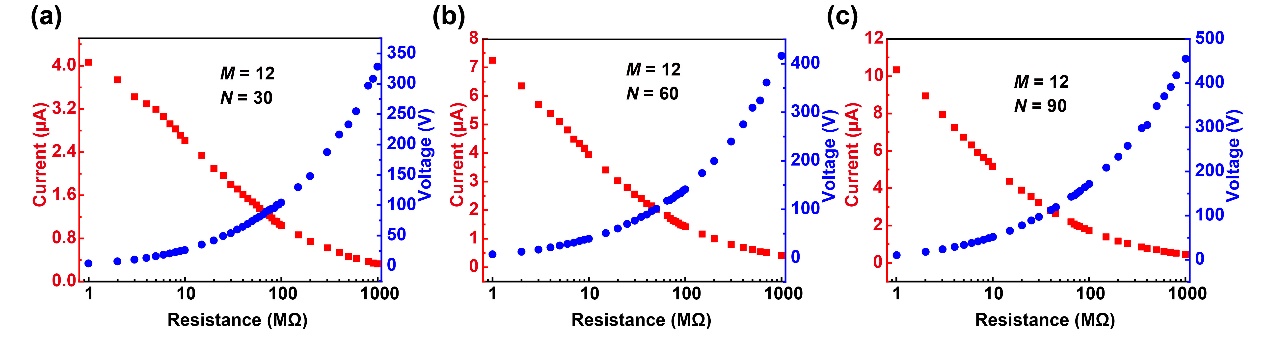


Figure S5. The output voltage and current for H-TENG with M=(a) 30, (b) 60, and (c) 90 with different load.


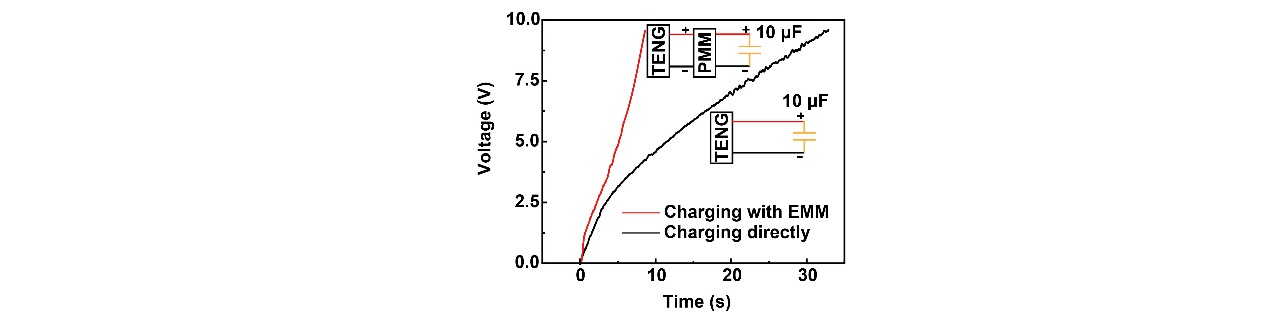


Figure S6. **Charging efficiency comparison by a H-TENG**

**Supporting Information video S1**. The LEDs are continuously illuminated by the R-TENG.

**Supporting Information video S2**. R-RMS simultaneously collects raindrop energy and measures rainfall for self-powered wireless rainfall monitoring and data transmission.
